# Allele Intersection Analysis: A Novel Tool for Multi Locus Sequence Assignment in Multiply Infected Hosts

**DOI:** 10.1371/journal.pone.0022198

**Published:** 2011-07-15

**Authors:** Wolfgang Arthofer, Markus Riegler, Hannes Schuler, Daniela Schneider, Karl Moder, Wolfgang J. Miller, Christian Stauffer

**Affiliations:** 1 Molecular Ecology Group, Institute of Ecology, University of Innsbruck, Innsbruck, Austria; 2 Hawkesbury Institute for the Environment, University of Western Sydney, Richmond, Australia; 3 Department of Forest and Soil Sciences, Institute of Forest Entomology, Forest Pathology and Forest Protection, Boku, University of Natural Resources and Life Sciences, Vienna, Austria; 4 Centre of Anatomy and Cell Biology, Medical University of Vienna, Vienna, Austria; 5 Department of Landscape, Spatial and Infrastructure Sciences, Institute of Applied Statistics and Computing, Boku, University of Natural Resources and Life Sciences, Vienna, Austria; Technion-Israel Institute of Technology, Israel

## Abstract

*Wolbachia* are wide-spread, endogenous α-Proteobacteria of arthropods and filarial nematodes. 15–75% of all insect species are infected with these endosymbionts that alter their host's reproduction to facilitate their spread. In recent years, many insect species infected with multiple *Wolbachia* strains have been identified. As the endosymbionts are not cultivable outside living cells, strain typing relies on molecular methods. A Multi Locus Sequence Typing (MLST) system was established for standardizing *Wolbachia* strain identification. However, MLST requires hosts to harbour individual and not multiple strains of supergroups without recombination. This study revisits the applicability of the current MLST protocols and introduces Allele Intersection Analysis (AIA) as a novel approach. AIA utilizes natural variations in infection patterns and allows correct strain assignment of MLST alleles in multiply infected host species without the need of artificial strain segregation. AIA identifies pairs of multiply infected individuals that share *Wolbachia* and differ in only one strain. In such pairs, the shared MLST sequences can be used to assign alleles to distinct strains. Furthermore, AIA is a powerful tool to detect recombination events. The underlying principle of AIA may easily be adopted for MLST approaches in other uncultivable bacterial genera that occur as multiple strain infections and the concept may find application in metagenomic high-throughput parallel sequencing projects.

## Introduction

*Wolbachia* are obligatory endosymbiotic α-Proteobacteria found in 15–75% of all insect species worldwide [1]–[4], in many other arthropods and filarial nematodes [5], [6]. The bacteria are usually transmitted by maternal inheritance and have developed sophisticated methods to manipulate host reproductive systems in order to increase the rate of infected female offspring. These alterations include cytoplasmic incompatibility (CI), thelytokous parthenogenesis, male killing and feminisation [7]–[11]. The wide range of infected species suggests an ability of *Wolbachia* to spread horizontally to new hosts [12], [13]. Their potential for horizontal transmission, combined with high maternal transmission efficiencies and low levels of endosymbiont loss by environmental curing [14], as well as the emergence of novel strains due to recombination [15]–[18] are expected to contribute to an accumulation of *Wolbachia* strains in individual hosts. Multiple infections by *Wolbachia* are commonly found: up to eight distinct strains have been isolated from individual hosts [19], and for 28 out of 111 species listed currently in the MLST database (see below) more than one *Wolbachia* strain is described. The fruit fly *Rhagoletis cerasi* is an established field model harbouring three A-group (*w*Cer1, *w*Cer2, *w*Cer4) and one B-group (*w*Cer5) *Wolbachia* in high titre [20]–[22], with hints of an A/B recombinant strain (*w*Cer3) in permanently low titre, traceable only by Southern transfer of PCR products and hybridization with a *wsp* specific probe [20].

*Wolbachia* have been assigned to eight supergroups, A to H, based on phylogenetic signals from the 16S rRNA gene and the genes *ftsZ* and *wsp* [5], [11], [23]–[25]. Most strains found in insects belong to supergroup A and B. Recombination within strains [16], between strains [15] and between supergroups [15], [11], [26] makes single gene typing approaches unsuitable for strain characterization, and for this reason, a Multi Locus Sequence Typing (MLST) system was introduced [27]. MLST is based on partial sequences of five ubiquitous housekeeping genes, *gatB*, *coxA*, *hcpA*, *ftsZ* and *fbpA*. The genes are amplfied using either standard primers amplifying strains from all supergroups or, alternatively, A- and B-group specific primers. The standard MLST protocol recommends direct sequencing of PCR products. In an online database (http://pubmlst.org/wolbachia/) a unique number is assigned to each identified allele so that any *Wolbachia* strain can be characterized by an individual, numeric code. The fast evolving and recombining *wsp* gene was not included, but serves as an optional typing marker for discrimination of closely related strains [28].

MLST for *Wolbachia* is a step forward in strain characterization but raises an important yet unresolved issue: the use of supergroup specific primers and direct sequencing is impractical in individuals that host more than one strain of the same supergroup due to ambiguous sequence reads [13]. Also, segregation of alleles by cloning and sequencing of plasmids does not resolve the assignment of alleles to individual strains. Thus, application of MLST is currently limited to individuals harbouring not more than one of each A- and B-group strain [13], [27].

Multiple infections can artificially be segregated by transinfection [22], [29], antibiotic treatment [30], [31] or cell culturing [32]. These techniques are technically challenging, laboursome and of uncertain outcome. In this study, we present a novel method, Allele Intersection Analysis (AIA), enabling correct assignment of MLST alleles retrieved from multiply infected individuals. AIA requires (a) the identification of the *Wolbachia* strains infecting a single individual (further referred to as ‘infection type’) using a highly variable marker gene and (b) the cloning and sequencing of MLST alleles from a pair of multiply infected individuals that share or differ in only one *Wolbachia* strain. By this, two sets of sequences, one from each individual, are generated. In the case of a shared *Wolbachia* strain, the allele found in both sets (the intersection) belongs to the shared strain; reciprocally, in the case that the pair differs in exactly one strain, its allele will be found exclusively in one set (the complement). Unresolved alleles are carried forward using other pairs of infection types until all the alleles have been assigned. Step (b) is performed independently for each MLST locus. Combinations of infection types that allow complete allele assignment are termed ‘informative’.

## Material and Methods

### Allele Intersection Analysis: Simulations

**Figure 1:**
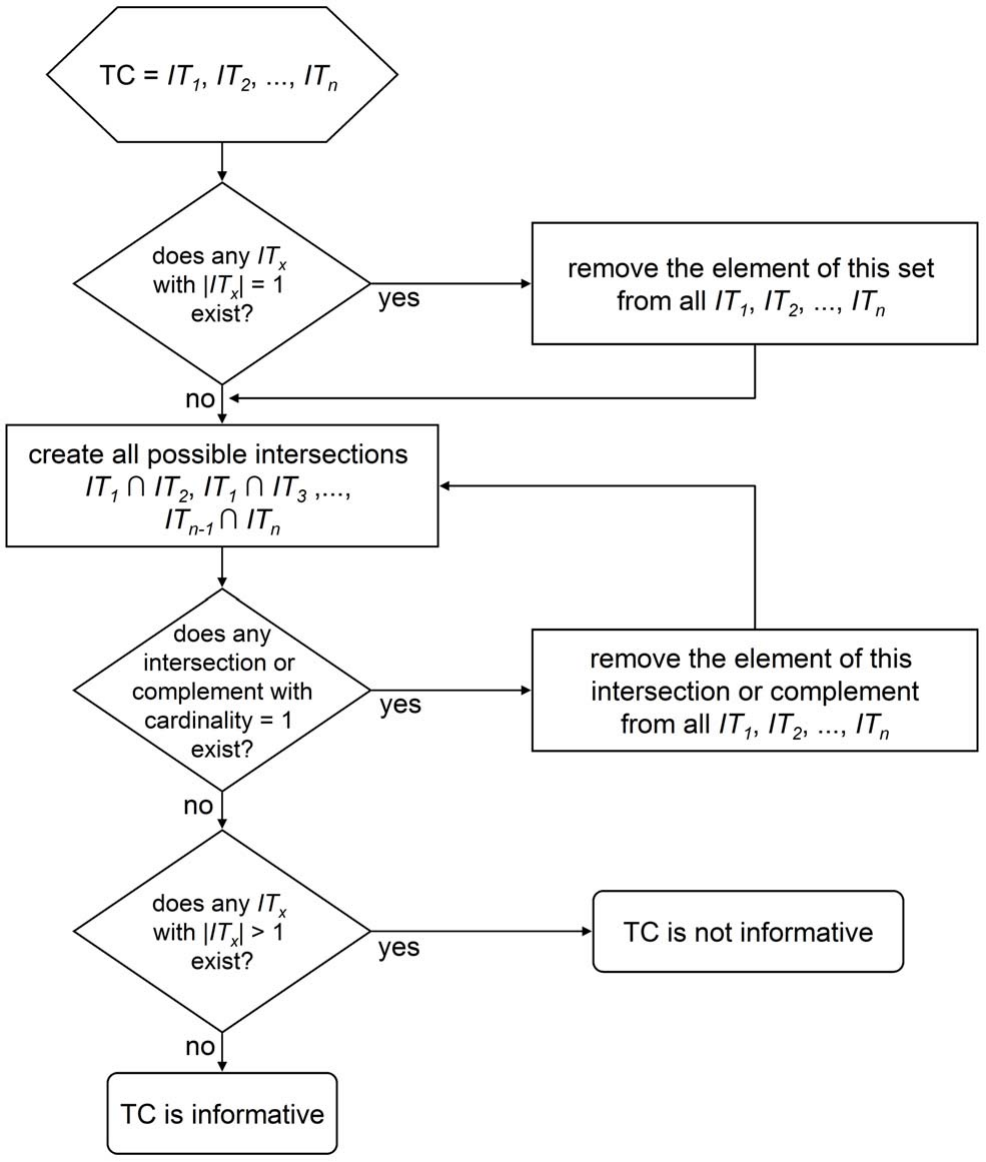
Test for informativeness. Each infection type is considered as a set with the *Wolbachia* strains as its elements. All sets are checked for having a cardinality of 1. If such a set is identified, the element contained in it subtracted from all sets. Next, all possible pairs of sets are explored for intersections or complements with a cardinality of 1. If found, the contained element is subtracted from all sets and the exploration of all possible remaining pairs is repeated until no intersection or complement with a cardinality of 1 can be identified. If the cardinality of the largest remaining set is >1, the type combination is not informative; otherwise the type combination is informative and allows complete allele assignment.

AIA depends on an informative combination of infection types. As the distribution pattern of informative among all possible type combinations does not follow simple mathematical equations, we simulated a species infected with *n* = [2 .. 7] *Wolbachia* strains and tested all possible combinations of *k*≤*n* infection types for informativeness. The test algorithm is outlined in figure 1: each infection type is considered as a set and the *Wolbachia* strains present in this infection type as the elements of the set. As a first step the entirety of all sets is checked for sets with a cardinality of 1 (a set containing only one element, equalling a single-infected individual; see Glossary S1 for a glossary on set theory terms). If such a set is identified, the element contained in it is considered fully resolved and removed from all sets, which are now termed ‘reduced sets’. Next, all possible pairs of (reduced) sets are explored for intersections (A ∩ B) or complements (A \ B and B \ A) with a cardinality of 1. If found, the contained element is removed from all reduced sets and the exploration of all possible remaining pairs is repeated. Two stop criteria will finish the loop of intersection or complement search and element removal: (a) no intersection or complement with a cardinality of 1 can be identified and the cardinality of the largest reduced set is still >1; in this case the type combination is not informative. (b) the cardinality of the largest reduced set drops to 1; in this case the type combination is informative and allows complete allele assignment. This algorithm is also implemented into the program TCinfo, which tests a manually entered combination. TCinfo is provided with the supplemental material of this paper (Computer Program S1: Windows® executable; Computer Program S2: source code).

To assess the power of AIA for detecting cryptic recombinations in which one complete MLST allele was exchanged between strains, we simulated a species infected with the *Wolbachia* strains A, B, C and R, assuming that R is a cryptic AxB recombinant that is misdiagnosed as A. An investigator not aware of the presence of strain R would screen this species for informative type combinations resolving A, B and C. Thus, we created all possible combinations that fulfil this criterion and searched them for combinations in which suspicious cloning results reveal the presence of R.

### Insect samples and determination of infection types

In a preceding study [20], DNA of 83 *R. cerasi* pupae from eight European regions (PL, CZ, AU, CH, IT-North, IT-Sicily East, IT-Sicily West, PT; Table 1) were extracted using the Sigma GenElute Mammalian DNA extraction Kit (Sigma) following the protocol of the manufacturer. DNA was eluted in 50 µl TE (10 mM Tris, 1 mM EDTA, pH = 8.0) and stored at −20°C. In addition, 24 adult flies were collected from yellow sticky traps in May 2008 in an Eastern Austrian location (Neufeld, Burgenland) where all identified *Wolbachia* strains are present; DNA was extracted analogously. Transinfected *C. capitata* (44) DNA extracts from lines WolMed88.6 containing *w*Cer2 and WolMedS10.3 containing *w*Cer4 were kindly provided by Kostas Bourtzis (University of Ioannina, Greece). The infection type of all samples was determined by amplification of the *wsp* gene with specific primers as described in [20].

**Table 1:** Distribution of infection types.

Location	n	Infection type (*w*Cer strain nr.)							
		1	1&2	1&4	1&5	1&2&4	1&2&5	1&4&5	1&2&4&5
Poland	4	1	1	1	0	1	0	0	0
Czech Republic	12	0	0	12	0	0	0	0	0
Austria	24	0	0	0	0	3	4	0	17
Switzerland	4	0	0	0	0	0	1	0	3
Italy – North	4	0	0	0	0	2	2	0	0
Italy – Sicily East	16	0	0	0	3	0	2	11	0
Italy – Sicily West	16	0	0	0	1	0	3	6	6
Portugal	3	0	0	0	0	0	0	3	0
**Total (pupae)**	**83**	**1**	**1**	**13**	**4**	**6**	**12**	**20**	**26**
**Neufeld (adult)**	**24**	**0**	**4**	**0**	**1**	**2**	**9**	**0**	**8**

Distribution of infection types in pupae from different European locations and in adult flies from Neufeld (AT). *w*Cer1 is fixed in all populations. *w*Cer2 and *w*Cer5 are absent in most north-eastern regions of Europe. The multitude of infection types at distinct locations in indicative for incomplete transmission of strains *w*Cer2, *w*Cer4 and *w*Cer5.

### Allele Intersection Analysis: Practical approach

Four individuals, representing different infection types, were selected for AIA (Table 2): the single infected samples WolMed88.6 (*w*Cer2) and WolMedS10.3 (*w*Cer4), a double infected fly from Znojmo (CZ) (*w*Cer1&4) and a triple infected fly from Horitschon (AT) (*w*Cer1&2&5).

**Table 2:** Insect samples for allele intersection analysis.

Sample name	Species	Origin	Infection type			
			*w*Cer1	*w*Cer2	*w*Cer4	*w*Cer5
WolMed 88.6	*C. capitata*	Transinfection		+		
WolMed S10.3	*C. capitata*	Transinfection			+	
Znojmo	*R. cerasi*	CZ	+		+	
Horitschon	*R. cerasi*	AT	+	+		+

Two single infected, one double infected and one triple infected individual of *R. cerasi* were used to assemble an informative type combination.

**Figure 2:**
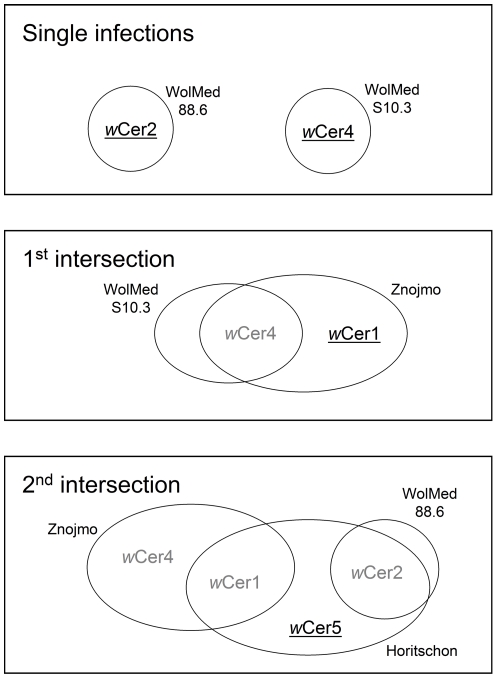
Application of allele intersection analysis in *R. cerasi*. Alleles identified in a current step are underlined. Alleles identified in earlier steps are grey. Single infection step: The alleles of *w*Cer2 and *w*Cer4 can be readily assigned as single infected individuals WolMed88.6 and WolMedS10.3 are available. 1^st^ intersection: All alleles in the double infected individual from Znojmo which are not *w*Cer4 must be *w*Cer1. 2^nd^ intersection: All alleles in the triple infected individual from Horitschon which are neither *w*Cer1 nor *w*Cer2 must be *w*Cer5. This procedure was performed individually for each MLST locus.

MLST PCR reactions were performed on a 2720 thermal cycler (Applied Biosystems) using 0.8 µl template DNA in a master mix containing 1× Mg-free buffer (Fermentas), 2 mM MgCl_2_, 100 µM dNTPs, 0.2 µM of each primer and 0.2 U Taq polymerase (Fermentas) with a total reaction volume of 10 µl. Standard MLST primers were used except for locus *fbpA*. The standard primers of this locus showed a weak amplification of *w*Cer5 when high amounts of A-group strains were present in the template, and lead to loss of B-group sequences after cloning. Thus, *fbpA* was additionally amplified with B-group specific primers. Primer sequences and cycling conditions were used as suggested in the MLST established protocols (http://pubmlst.org/wolbachia). PCR products were purified with the peqGold Cycle Pure Kit (peqlab), eluted in 10 µl sterile water, and a 0.8 µl aliquot was ligated into the pTZ57R/T vector of the InsTAclone PCR cloning kit (Fermentas) according to the instructions of the manufacturer. The ligated plasmids were used for transformation of competent JM109 *E. coli* cells. For each individual and locus, 24 white colonies were picked, grown over night and plasmid DNA was extracted by alkaline lysis [33]. Insert size was determined by PCR with M13 forward and reverse primers and plasmids with correct insert sizes were Sanger sequenced by a commercial provider. Sequences were aligned with ClustalX [34], and based on the alignments the intersection steps for AIA were performed manually (Figure 2).

### Strain bias

To determine whether standard MLST primers exhibit strain bias, DNA of a single female fly collected in Neufeld (AT) with confirmed quadruple infection was analyzed. Quantification of *w*Cer load was carried out on a Rotorgene-Q PCR system (Qiagen) using 1× Qiagen SYBR Green Mastermix, 0.2 µl of strain specific *wsp* primers [20] and 1 µl template DNA in 10 µl total volume. Dilution series of plasmids carrying *w*Cer1, *w*Cer2, *w*Cer4 and *w*Cer5 *wsp* inserts with a concentration range from 10^1^ to 10^−3^ ng/µl served as standard. The cycling scheme was 3 min initial denaturation at 95°C followed by 45 cycles of 95°C (10 sec), 55°C (15 sec) and 72°C (40 sec), and a melting curve acquisition from 60°C to 95°C with 0.2°C step width.

MLST PCR reactions and amplicon purification were performed as described above. All standard and B-group specific primers were used. PCR of *wsp* was carried out with primers *wsp*-81F/*wsp*-691R (unspecific) [23] and *wsp*-81F/*wsp*-522R (B-group specific) following the protocol in reference 25.

Cloning of purified PCR products was performed as described above, and after overnight growth 16 white colonies per ligation were picked for plasmid extraction. Plasmids with correct insert sizes were Sanger sequenced by a commercial provider. Based on the retrieved sequences, each plasmid was assigned to the corresponding strain. For each *Wolbachia* strain and MLST locus, the expected number of plasmids and the relative deviation between expected and observed plasmid frequency was calculated based on the relative strain load.

## Results

### Simulations

The number of possible infection types in an *n*-fold infected species is given by
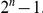
The grouping of *k*≤*n* types allows a maximum possible number of type combinations equalling
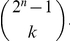
Any type combination will be informative when *k*>*n*. The smallest *k* to provide an informative combination was two for *n* = [2,3], three for *n* = [4,6] and four for *n* = 7 (Table 3). The rate of informative combinations increases fast when *k* approaches *n*. Considering all *k* and *n*>2 tested, the rate of informative combinations lies between 60% (*n* = 4) and 80% (n = 7). Predictions for *n*>7 are constrained by available computing power. Figure S1 shows two examples of informative type combinations created in the simulation, and the intersections needed to assign all *Wolbachia* alleles to the correct strain.

**Table 3:** Rate of informative type combinations.

n	2			3			4			5			6			7		
ITs	3			7			15			31			63			127		
k	iTC	TC	iTC/TC	iTC	TC	iTC/TC	iTC	TC	iTC/TC	iTC	TC	iTC/TC	iTC	TC	iTC/TC	iTC	TC	iTC/TC
2	3	3	1.00	3	21	0.14	0	105	0.00	0	465	0.00	0	1953	0.00	0	8001	0.00
3	-	-	-	32	35	0.91	140	455	0.31	420	4495	0.09	840	39711	0.02	0	33375	0.00
4	-	-	-	-	-	-	1015	1365	0.74	13965	31465	0.44	148050	595665	0.25	1347360	10334625	0.13
5	-	-	-	-	-	-	-	-	-	126651	169911	0.75	3970806	7028847	0.56	106389990	254231775	0.42
6	-	-	-	-	-	-	-	-	-	-	-	-	53354350	67945521	0.79	3494224062	5169379425	0.68
7	-	-	-	-	-	-	-	-	-	-	-	-	-	-	-	74698954066	89356415775	0.84
**total**	3	3	1.00	35	56	0.63	1155	1925	0.60	141036	206336	0.68	57474046	75611697	0.76	78300915478	94790402976	0.83

Exhaustive search for informative type combinations. All possible combinations of *k* infection types and *n Wolbachia* strains with *k*≤*n* were generated and tested for informativeness. The ratio of informative type combinations increases fast when *k* approaches *n*.

n .. number of *Wolbachia* strains, ITs .. maximum number of possible infection types, k .. number of ITs in type combination, iTC .. number of informative type combinations, TC .. total number of possible type combinations.

The simulation of an A, B, C and R infected species resulted in 560 possible combinations of 2 and 3 infection types (Table S1). Due to the chosen diagnostic system the cryptic AxB recombinant R is misdiagnosed as A, and 299 type combinations fulfil the criterion of being informative for A, B, and C in the alleged triple-infected species. 235 (78.6%) of them would create suspicious cloning results, as alleles would be present that can not be explained by the outcome of the diagnostic procedure.

### Presence of different infection types in individual insects

**Figure 3:**
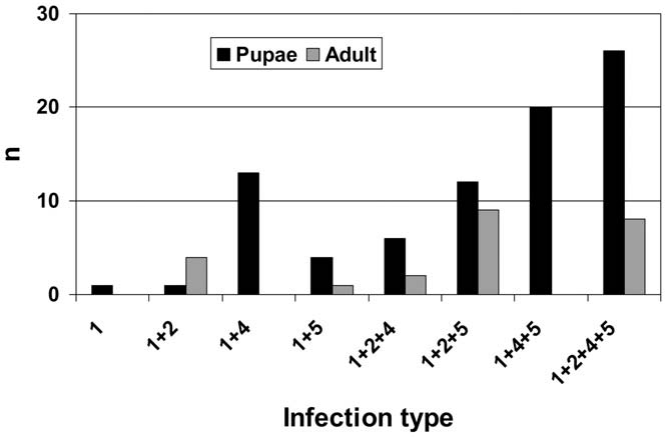
Infection type frequencies in *R. cerasi* field populations. Frequency of different infection types in 83 pupae and 24 adult flies. Pupae were collected from eight European regions, while all adult flies were collected at a single location in eastern Austria. Strain *w*Cer1 is in fixation at all collection sites. All other strains are found only some individuals, indicative for incomplete transmission. Patterns like these allow the identification of complete MLST profiles without need of further physical strain segregation.

AIA requires sufficiently diverse *Wolbachia* within a multiply infected species so that an informative type combination may be detected. This involves the diagnosis of *Wolbachia* from individuals. Of the four high titre strains infecting *R. cerasi*, *w*Cer1 is fixed in all populations [20], [21]. Assuming that the other three strains have not reached fixation in all geographic regions and/or individuals there are eight possible infection types in combination with *w*Cer1. Indeed, all possible types were found in the 83 pupae sampled from all over Europe (Figure 3). The 24 adult flies collected from Neufeld (AT), a location where all *w*Cer strains are present, resembled five different infection types (Figure 3), indicative for incomplete transmission of *w*Cer2, *w*Cer4 and *w*Cer5. The local distribution of infection types was biased according to the geographic distribution of the *w*Cer strains (Table 1). Higher degree infections were generally overrepresented.

### Allele intersection analysis AIA

We created an informative set of infection types, allowing unambiguous assignment of all alleles to distinct strains, by combining two singly, one doubly and one triply infected insect (Table 2). The available singly infected specimens that resulted from previous transinfection experiments into *Wolbachia* free *C. capitata* [22], [35], simplified the process of allele intersection by reducing the cloning effort and allowed the direct identification of *w*Cer2 and *w*Cer4 MLST alleles. A first intersection (Figure 2) was made between the known sequences of *w*Cer4 and the set of sequences derived from cloning the *w*Cer1 & *w*Cer4 doubly infected fly from Znojmo, resolving the alleles of strain *w*Cer1. In a second intersection, the already defined sequences of *w*Cer1 and *w*Cer2 were compared to the clones retrieved from the *w*Cer1 & *w*Cer2 & *w*Cer5 triply infected host from Horitschon, and the unique sequences from this host were assigned to *w*Cer5. After two intersections, MLST of the four *R. cerasi* strains was fully resolved. All *w*Cer strains showed unique alleles for all MLST loci except for the *coxA* allele shared by *w*Cer1 and *w*Cer4. All identified alleles were cross-checked with and new alleles submitted to the MLST database. A summary of the allele IDs is shown in table 4.

**Table 4:** MLST characterization of *w*Cer.

Strain	HVR1	HVR2	HVR3	HVR4	ST	gatB	coxA	hcpA	ftsZ	fbpA
***w*** **Cer1**	1	12	21	144	158	8	**84**	**103**	**79**	**160**
***w*** **Cer2**	1	12	21	19	13	1	1	1	3	1
***w*** **Cer4**	67	77	12	9	159	53	**84**	85	70	79
***w*** **Cer5**	69	17	3	23	160	101	**85**	40	22	4

HVR alleles, sequence types and allele IDs of *Wolbachia* strains infecting *R. cerasi*; new alleles are printed in bold.

### Strain bias

Quantitative PCR of the *wsp* locus from one quadruply infected *R. cerasi* female revealed a strain load ratio of *w*Cer1∶*w*Cer2∶*w*Cer4∶*w*Cer5 = 0.49∶1∶0.45∶0.09. We sequenced 148 plasmids cloned from *wsp* and MLST PCR products from the same specimen showing correct insert sizes. Nine plasmids (6.1%), all derived from the loci *wsp* and *fbpA*, showed unique recombinant sequences not reproducible in repeated reactions. They were therefore considered as PCR artefacts and excluded from further analysis. Observed and expected numbers of plasmids carrying the different *w*Cer sequences are given in table S2.

**Figure 4:**
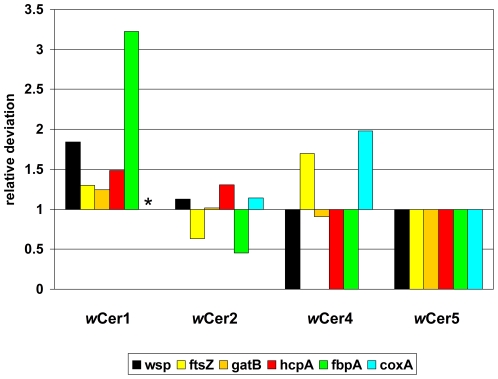
MLST primer bias. Relative deviations between expected and observed plasmid frequencies. Standard MLST primers were used to clone plasmids from a quadruply infected host. Expected plasmid frequencies were calculated based on the relative load of each *Wolbachia* strain, assuming no primer bias, and compared with the observed plasmid frequencies. At a value of 1, expected and observed frequency are identical. Values higher than 1 indicate that a strain was cloned more often than expected. A value of 0 indicates that the corresponding strain was not found in any plasmid. (*) *coxA* not applicable in *w*Cer1, as the allele is shared with *w*Cer4.

Figure 4 shows the relative deviations between expected and observed plasmid frequencies for the standard primers. *w*Cer1 is in average 1.82-fold overrepresented, while only 0.76 times the expected number of *w*Cer4 plasmids was found; plasmids carrying *wsp*, *hcpA* and *fbpA* were missing for *w*Cer4. Based on its contribution to the total *Wolbachia* load, 6.1 of 135 plasmids should originate from *w*Cer5, but not a single such plasmid was observed.

The specifity of B-group primers was confirmed for *coxA*, *gatB*, *ftsZ* and *hcpA*, but disproved for locus *fbpA*, where 50% of the plasmids carried inserts from the A-group strains *w*Cer1, *w*Cer2 and *w*Cer4.

## Discussion

Hosts can be infected by a multitude of *Wolbachia* strains rendering the assignment of MLST alleles a challenge. So far, acquisition of MLST sequence types from a multiply infected host was possible only if individual specimens were singly infected, or harboured one A- and B-group strain each [13], [27]. A combination of host individuals that are multiply and differently infected, i.e. carry different infection types, can provide inference on strain specific MLST profiles. Through a series of operations, AIA tests whether the combination fulfils criteria of informativeness. To evaluate the practical applicability of the theory behind AIA, it was first determined whether informative combinations of infection types are exceptional or frequent in field populations. Our simulations indicate that the majority of all possible type combinations is informative, and several studies suggest that the differences of *Wolbachia* distribution in field sampled insects are sufficient to apply AIA in most cases of multiply infected species [16], [20], [29], [36], [37].

### Application of AIA in the field model species R. cerasi

The multiply infected field model species *R. cerasi* is refractory to MLST characterisation by conventional means: it harbours three *Wolbachia* strains of supergroup A, with one of these strains in fixation in all populations sampled so far, one supergroup B strain and traces of one recombinant strain. By applying AIA, a complete characterization of four *Wolbachia* strains infecting *R. cerasi* was achieved using cloned PCR products originating from four individual flies of which two were multiply infected with two A-group strains, and two one A- plus one B-group strain, respectively. Availability of single-infected individuals from an artificial *Wolbachia* microinjection reduced cloning effort, but even if these single infections were not available, the set of identified infection types in the 83 pupae used in this study would have allowed the assembly of 81 different informative type combinations (Table S3): 20 out of 56 possible type combinations of three individuals with unique infection types are informative; combining four individuals, 61 of the 70 possible combinations are informative. Figure S2 shows an example of an alternative informative set using three *R. cerasi* individuals, one doubly (two A-group strains) and two triply-infected (two A- and one B-group strains each).

### Required sequencing effort

Assessment of *Wolbachia* diversity in a population requires cloning and sequencing of PCR products, and the choice of an insufficient number of plasmids may leave some diversity undetected. In a preceding study [20], rarefaction analysis [38] was shown as an efficient tool to determine whether a sufficient number of sequences was analysed for comprehensive assessment of strain diversity.

Sequencing effort to identify the *Wolbachia* strains of the individuals forming the type combination is more relaxed, as the absolute number of strains present in each probed sample was already determined by strain specific PCR. Thus, the number of alleles at a given MLST locus should be equal to or lower than the number of *Wolbachia* strains. A smaller number of alleles than strains allows two interpretations: (a) two strains share the same allele for a given locus [27] or (b) a strain is exhibiting a null allele due to insufficient PCR amplification [39]. To safeguard against case (b), repetition of cloning using alternative MLST primers (e.g. ∼64fold degenerate primers; http://pubmlst.org/wolbachia/info/amp_seq_single.shtml) can be performed. Alternatively, (a) can be proven by successfully isolating the same allele from two individuals that do not share the questioned strain; this approach was used here to prove the identity of *coxA in* wCer1 and wCer4: the same allele was isolated from WolMedS10.3 (wCer4) and Horitschon (wCer1&2&5).

In some cases, cloning of a MLST locus might reveal more alleles than strains predicted by diagnostic PCR. If not caused by PCR artefacts (see [40] for a general discussion on PCR error, and [41] for *in vitro* recombination during PCR), such a finding indicates either insufficient sampling when assessing the host species' *Wolbachia* community, or a shared allele in the diagnostic marker. If PCR artefacts can be excluded, the MLST locus exhibiting the additional allele has to be established as additional diagnostic marker, and the number of strains present in the species has to be corrected upwards.

### Potential impact of recombination

Recombination is frequent in *Wolbachia*, and large genomic regions may be exchanged between strains [15], [28]. Recombination events may impact AIA at two points: (a) within the diagnostic marker locus and (b) between MLST loci. In case of (a), the diagnostic PCR will overestimate strains than alleles can be identified from the MLST clones; this scenario resembles a generalized case of shared MLST alleles, which can be handled as described before.

**Figure 5:**
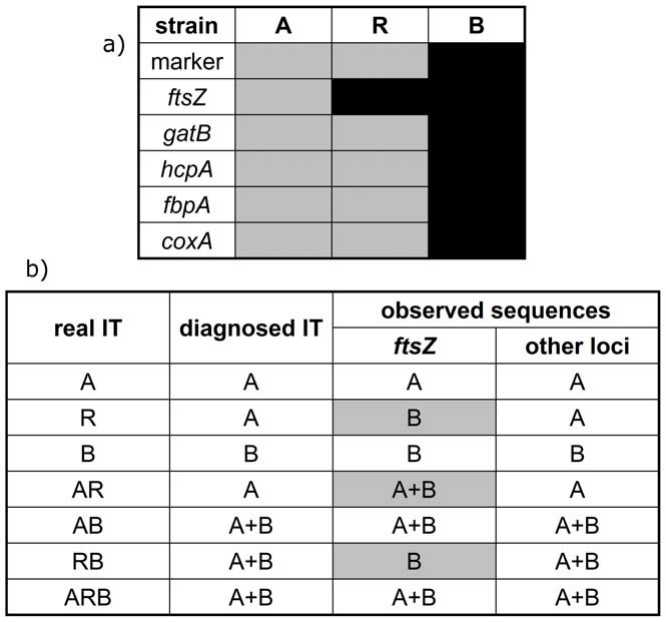
Recombination detection with AIA. (a) In a hypothetical recombination event, the *ftsZ* locus of strain B has invaded into strain A, forming the new strain R. The diagnostic marker used for infection type (IT) identification will mistype R as A. (b) Cloning results from different ITs. In three cases (shaded), suspicious sequences will be observed.

In scenario (b), at least one complete MLST locus has moved from one *Wolbachia* strain to another, leaving the diagnostic marker unchanged. Figure 5a illustrates such a recombination between two strains A and B at locus *ftsZ*, creating the new strain R. Diagnostic PCR will mistype this strain as A and suggest a double-infected species, while effectively three strains are present. A triple infected species allows seven possible infection types (Figure 5b). The cloning efforts for AIA would generate suspicious patterns in the three infection types where the recombinant strain has segregated from at least one of its parents, revealing the hidden recombination: the single R infection directly shows the recombination; the combination A+R, diagnosed as single infection with strain A, will present two alleles for *ftsZ*; finally, the combination R+B, diagnosed as A+B, will not show any allele of strain A in the *ftsZ* clones.

We have simulated a more complex situation with three identified strains and one cryptic recombinant, and have shown that 78.6% of the possible type combinations would create suspicious cloning results, uncovering the cryptic strain. Resolving recombinant *Wolbachia* strains will remain a complex issue and is only possible if the recombinant has segregated from at least one parent, either by natural processes like incomplete transmission, or by artificial transinfection. If segregation has occurred, application of AIA will detect the strain with a high likelihood, which can be improved by adding redundancy to the approach, i. e. by cloning alleles of more individuals than needed for a minimal informative type combination. As recombination events between supergroups exist [26], it is important to combine the sequences retrieved with standard and group-specific primers to one alignment before applying AIA.

### Strain bias

Isolation of a specific allele by cloning of PCR products does not only depend on its density in the template, but also in the intensity of amplification compared to that of competing alleles present in the reaction. End-point PCR from mixed templates is often assumed to be a semiquantitative amplification method, where the ratio of generated amplicon after a fixed number of cycles resembles the template ratio in the original mixture [41]. This assumption is challenged by numerous factors affecting PCR such as lack of primer specificity and formation of secondary template structures. The possible bias may span from 1∶1 amplicon formation irrespective of the initial template ratio [42] to complete suppression of a minor template in presence of a more frequent one [43]. Biased amplification of individual loci was reported for MLST of some strains of *Pseudomonas aeruginosa* [44] and *Clostridium difficile* [39].

The comparison of strain load determined by qPCR and plasmid frequencies of cloned MLST amplicons indicates that standard MLST primers underlie strong strain bias (Figure S2, Table S2). Most striking is the fact that the standard primers for *hcpA* and *fbpA* (and the 81F/691R primers for *wsp*) failed to produce a single clone from strain *w*Cer4, which contributes to 22.2% of the original *Wolbachia* load, and that all standard primers did not amplify strain *w*Cer5. While it is evident that standard primers do amplify *w*Cer4 when present as a single infection [20], this proof is missing for *w*Cer5, as no individuals harbouring only this strain are currently available. Therefore it remains unclear whether the B-group origin of *w*Cer5 or its comparatively low contribution of 4.4% to the *Wolbachia* load are responsible for the absent amplification. It is also notable that MLST primers do not simply exhibit suppression of minor templates: *w*Cer1, with 24.1% load comparable to *w*Cer4, had elevated plasmid frequencies for all investigated loci. For an exhaustive detection of genotypes in multiply infected hosts both standard and group specific primers should be applied, ideally on individuals with different infection types.

The cloning approach revealed two more observations relevant for *Wolbachia* MLST. First, B-group specific primers for loci *coxA* and *fbpA* co-amplified a substantial proportion of A-group strains. It must therefore be doubted that group specific primers alone are sufficient for *in vitro* sequence segregation, especially when the A-B-group ratio is heavily biased towards one group. Direct sequencing of PCR products amplified with B-group specific primers from multiply infected *R. cerasi* samples repeatedly resulted in noisy electropherograms and erroneous base calls (data not shown), suggesting that cloning should be preferred to direct sequencing of PCR products, even if a supergroup contributes only one strain to a multiply infected host. Second, *in vitro* recombination [45] was observed in 16.7% of the plasmids cloned from *fbpA* standard and 21.4% from *fbpA* B-group specific primers (Table S1). Typical for *in vitro* rearrangements, the chimeras were not reproducible in independent replicas of PCR and cloning, demonstrating the necessity of at least two independent PCR reactions to confirm a novel genotype.

MLST is currently revolutionizing the way of typing *Wolbachia* infections, detecting evolutionary events and retrieving phylogenetic information of this endosymbiont [28]. So far, MLST was built on the assumption of singly infected systems or systems where one A- and B-group strain simultaneously infected a host without recombination – an assumption that is not realistic in the light of the high abundance of multiply infected *Wolbachia* host species [16], [19], [36], [37]. The AIA approach presented here is a novel and straightforward tool to apply MLST in multiply infected *Wolbachia* host species that were so far refractory to MLST typing. The method will render artificial strain segregation unnecessary in most cases, and highly reduce segregation effort in those where informative type combinations can not be found readily in natural field populations. Furthermore, AIA is a powerful tool to detect recombination events. We expect that AIA will improve allele assignment of *Wolbachia* MLST loci, and facilitate the research on the evolution, dynamics and population genetics of multiple infections in field hosts, rather than microbially streamlined lab hosts of *Wolbachia*. The underlying principle of AIA may easily be adopted for MLST approaches in other uncultivable bacterial genera that occur as multiple strain infections. It may also be useful for metagenomic sequencing projects [46], [47] that currently face difficulties with assembly of reads in multi-genome scenarios [48]. The assignment and assembly of metagenomic data from a parallel-sequencing approach of samples containing different type combinations could be supported by principles of AIA. In the case of multiply *Wolbachia* infected hosts this would involve the sequencing of a series of pools with distinct infection types, and then assigning contigs to the different *Wolbachia* genomes by using AIA like strategies in a parallel tagged pyrosequencing approach [49].

## Supporting Information

Figure S1 **Examples of informative type combinations.** Two examples of simulated, informative type combinations and the stepwise allele assignment of AIA. Each circle and capital letter reresents one individual, each strain is represented by a colored ellipse; alleles identified in earlier steps are dashed. S1.1: Five strains in three triple infected individuals. Step 1: the intersection A ∩ B resolves the red allele. Step 2: the intersection A ∩ C resolves the green allele; in the same step, the complements resolve the brown and pink allele. Step 3: The blue allele is resolved by removing the already identified red and pink alleles from the alignment of B. S1.2: Seven strains in four individuals; alleles identified in earlier steps are dashed. Step 1: alleles from A that are not found in D must belong to purple. Step 2: alleles in the intersect A ∩ B which are not purple belong to yellow. Step 3: alleles in the intersect A ∩ C which are not purple belong to yellow. Step 4: all yet unidentified alleles in A are blue. Step 5: alleles in the intersect B ∩ C which are not purple belong to brown. After identification of the brown allele, all unidentified alleles in B are pink, and unidentified alleles C are red.
(TIF)

Figure S2 **An alternative type combination for AIA in** ***R. cerasi*** **.** One out of 81 informative type combinations in *R. cerasi* that does not rely on artificial strain segregation: one doubly (A) and two triply (B, C) infected individuals allow the assignment of all alleles after two intersections. Alleles identified in a current step are underlined. Alleles identified in earlier steps are grey.
(TIF)

Table S1 **In a species diagnosed as triple infected with strains A, B, C, a cryptic recombinant R has formed.** The table shows all possible combinations of 2 and 3 infection types. Types leading to suspicious cloning results are shaded. Each combination is tested for informativeness to resolve A, B and C under the assumption that R is consequently mis-diagnosed as A.
(DOC)

Table S2 **Observed numbers of plasmids cloned from DNA of a quadruply infected host Neufeld, and expected numbers based on each strain's relative load in the original DNA extract.**
(DOC)

Table S3 **All possible 3- and 4-fold combinations of infection types found in** ***R. cerasi*** **field samples.** These is a total of 126 combinations, of which 81 are informative. Uninformative combinations are shaded.
(DOC)

Computer Program S1 **TCinfo is a small computer program that implements the test algorithm for informativeness described in** **figure 1** **.** File TCinfo.exe is a Windows® executable.
(EXE)

Computer Program S2 **File TCinfo.pas is an ASCII text file containing the source code of TCinfo.** The program was written in Free Pascal (http://www.freepascal.org) under the GNU General Public License as published by the Free Software Foundation.
(PAS)

Glossary S1 **Short descriptions of set therory terms used in this paper.**
(DOC)
