# Supplementary figures and images for: Allele Intersection Analysis: A Novel Tool for Multi Locus Sequence Assignment in Multiply Infected Hosts
Source: PLoS One. 2011 Jul 15;6(7):e22198. doi: 10.1371/journal.pone.0022198 (PMC3137623; doi:10.1371/journal.pone.0022198)

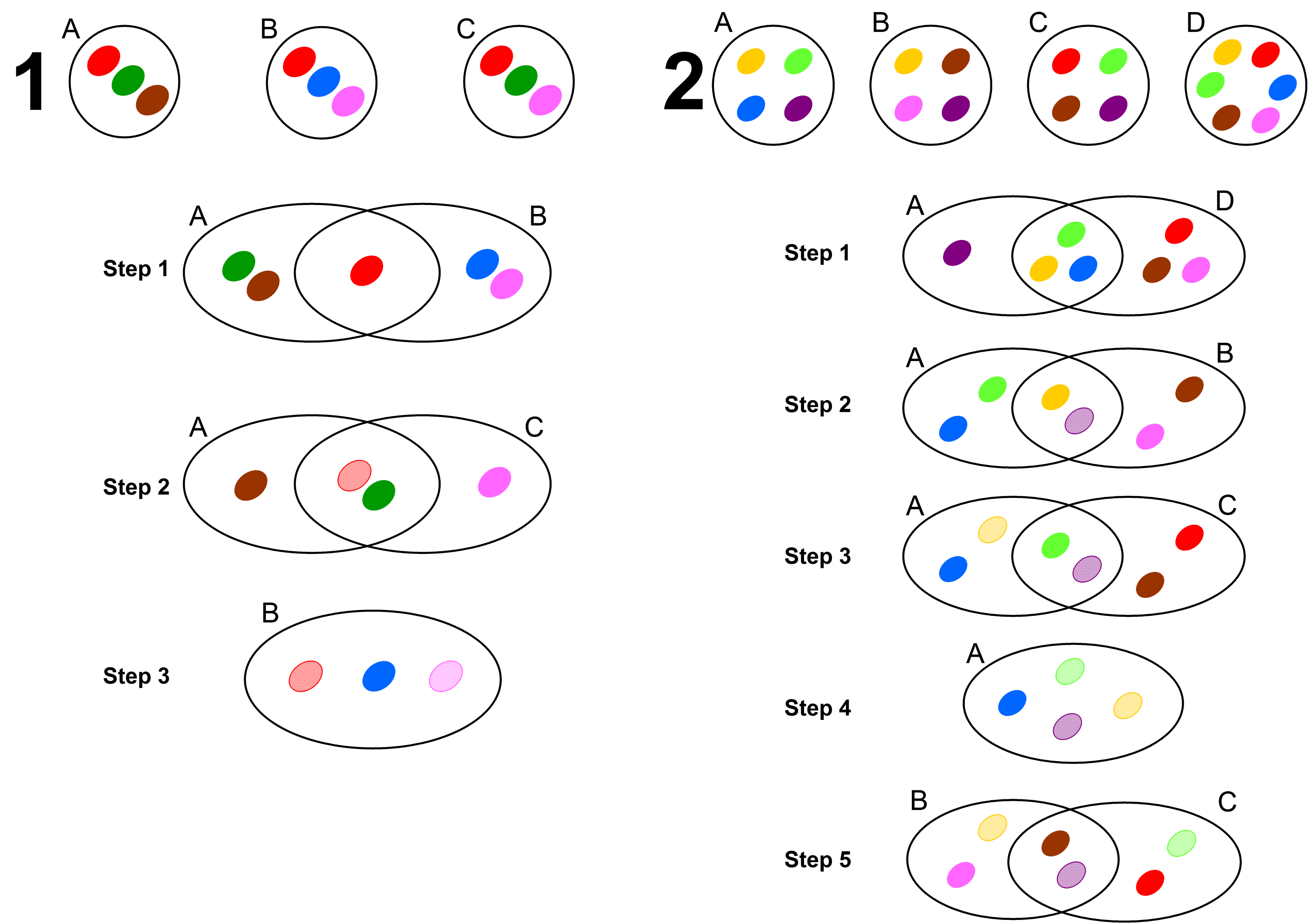

Supplement: Figure S1 — Examples of informative type combinations. Two examples of simulated, informative type combinations and the stepwise allele assignment of AIA. Each circle and capital letter reresents one individual, each strain is represented by a colored ellipse; alleles identified in earlier steps are dashed. S1.1: Five strains in three triple infected individuals. Step 1: the intersection A ∩ B resolves the red allele. Step 2: the intersection A ∩ C resolves the green allele; in the same step, the complements resolve the brown and pink allele. Step 3: The blue allele is resolved by removing the already identified red and pink alleles from the alignment of B. S1.2: Seven strains in four individuals; alleles identified in earlier steps are dashed. Step 1: alleles from A that are not found in D must belong to purple. Step 2: alleles in the intersect A ∩ B which are not purple belong to yellow. Step 3: alleles in the intersect A ∩ C which are not purple belong to yellow. Step 4: all yet unidentified alleles in A are blue. Step 5: alleles in the intersect B ∩ C which are not purple belong to brown. After identification of the brown allele, all unidentified alleles in B are pink, and unidentified alleles C are red. (TIF) [file pone.0022198.s001.tif]

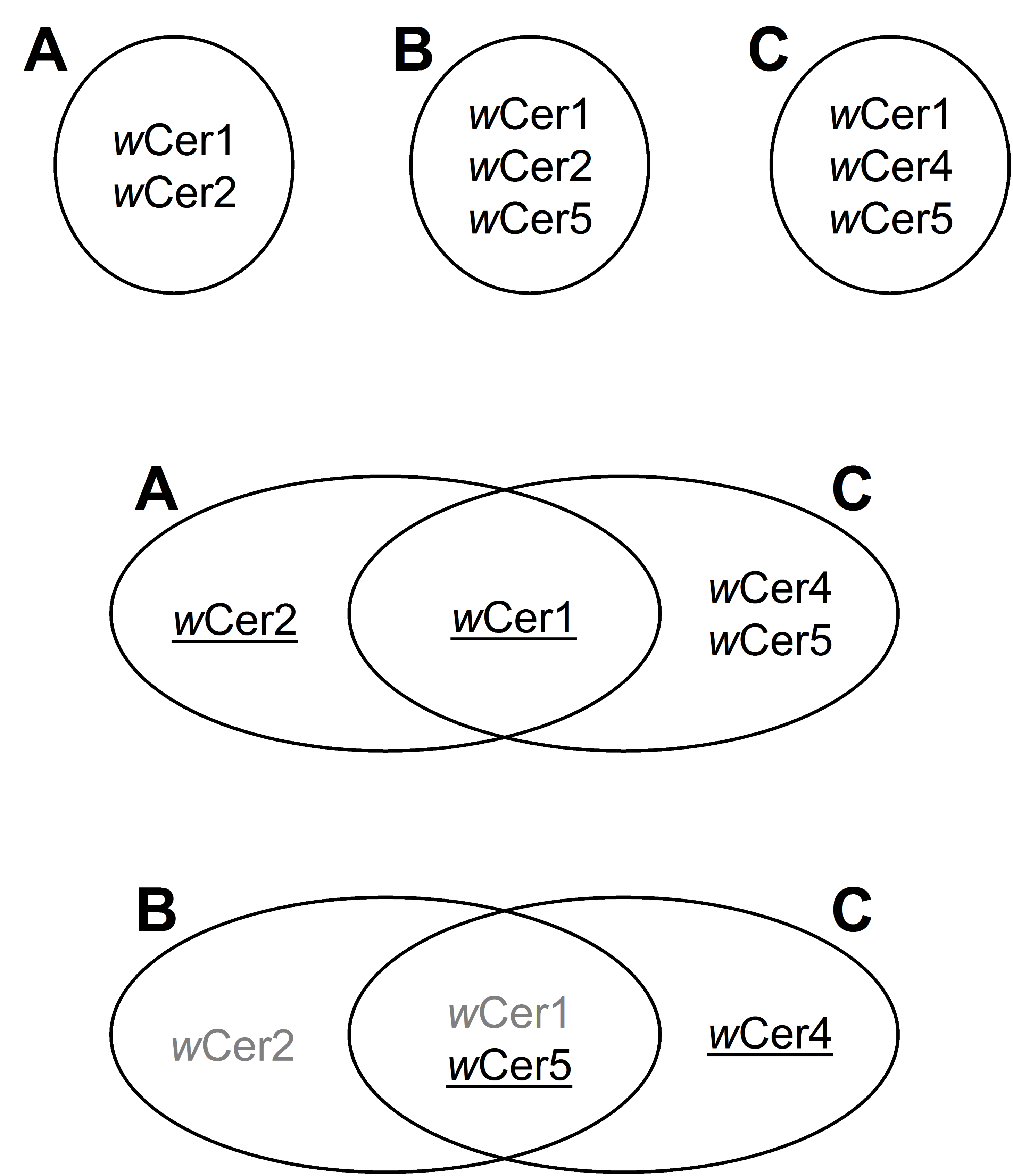

Supplement: Figure S2 — An alternative type combination for AIA in R. cerasi . One out of 81 informative type combinations in R. cerasi that does not rely on artificial strain segregation: one doubly (A) and two triply (B, C) infected individuals allow the assignment of all alleles after two intersections. Alleles identified in a current step are underlined. Alleles identified in earlier steps are grey. (TIF) [file pone.0022198.s002.tif]
