# Supplementary material for: Allele Intersection Analysis: A Novel Tool for Multi Locus Sequence Assignment in Multiply Infected Hosts
Source: PLoS One. 2011 Jul 15;6(7):e22198. doi: 10.1371/journal.pone.0022198 (PMC3137623; doi:10.1371/journal.pone.0022198)
Supplement: Table S1 — In a species diagnosed as triple infected with strains A, B, C, a cryptic recombinant R has formed. The table shows all possible combinations of 2 and 3 infection types. Types leading to suspicious cloning results are shaded. Each combination is tested for informativeness to resolve A, B and C under the assumption that R is consequently mis-diagnosed as A. (DOC) [file pone.0022198.s003.doc]

**Table S1**

In a species diagnosed as triple infected with strains A, B, C, a cryptic recombinant R has formed. The table shows all possible combinations of 2 and 3 infection types. Types leading to suspicious cloning results are shaded. Each combination is tested for informativeness to resolve A, B and C under the assumption that R is consequently mis-diagnosed as A.

| **#** | **IT1** | **IT2** | **IT3** | **contains R** | **suspicious cloning result** | **informative for ABC** | **suspicious and informative** |
| --- | --- | --- | --- | --- | --- | --- | --- |
| 1 | A | B | - |  |  |  |  |
| 2 | A | C | - |  |  |  |  |
| 3 | A | R | - | + | + |  |  |
| 4 | A | AB | - |  |  |  |  |
| 5 | A | AC | - |  |  |  |  |
| 6 | A | AR | - | + | + |  |  |
| 7 | A | BC | - |  |  |  |  |
| 8 | A | BR | - | + | + |  |  |
| 9 | A | CR | - | + | + |  |  |
| 10 | A | ABC | - |  |  |  |  |
| 11 | A | ABR | - | + |  |  |  |
| 12 | A | ACR | - | + | + |  |  |
| 13 | A | BCR | - | + | + |  |  |
| 14 | A | ABCR | - | + |  |  |  |
| 15 | B | C | - |  |  |  |  |
| 16 | B | R | - | + | + |  |  |
| 17 | B | AB | - |  |  |  |  |
| 18 | B | AC | - |  |  |  |  |
| 19 | B | AR | - | + | + |  |  |
| 20 | B | BC | - |  |  |  |  |
| 21 | B | BR | - | + | + |  |  |
| 22 | B | CR | - | + | + |  |  |
| 23 | B | ABC | - |  |  |  |  |
| 24 | B | ABR | - | + |  |  |  |
| 25 | B | ACR | - | + | + |  |  |
| 26 | B | BCR | - | + | + |  |  |
| 27 | B | ABCR | - | + |  |  |  |
| 28 | C | R | - | + | + |  |  |
| 29 | C | AB | - |  |  |  |  |
| 30 | C | AC | - |  |  |  |  |
| 31 | C | AR | - | + | + |  |  |
| 32 | C | BC | - |  |  |  |  |
| 33 | C | BR | - | + | + |  |  |
| 34 | C | CR | - | + | + |  |  |
| 35 | C | ABC | - |  |  |  |  |
| 36 | C | ABR | - | + |  |  |  |
| 37 | C | ACR | - | + | + |  |  |
| 38 | C | BCR | - | + | + |  |  |
| 39 | C | ABCR | - | + |  |  |  |
| 40 | R | AB | - | + | + |  |  |
| 41 | R | AC | - | + | + |  |  |
| 42 | R | AR | - | + | + |  |  |
| 43 | R | BC | - | + | + |  |  |
| 44 | R | BR | - | + | + |  |  |
| 45 | R | CR | - | + | + |  |  |
| 46 | R | ABC | - | + | + |  |  |
| 47 | R | ABR | - | + | + |  |  |
| 48 | R | ACR | - | + | + |  |  |
| 49 | R | BCR | - | + | + |  |  |
| 50 | R | ABCR | - | + | + |  |  |
| 51 | AB | AC | - |  |  | + |  |
| 52 | AB | AR | - | + | + |  |  |
| 53 | AB | BC | - |  |  | + |  |
| 54 | AB | BR | - | + | + |  |  |
| 55 | AB | CR | - | + | + | + | + |
| 56 | AB | ABC | - |  |  |  |  |
| 57 | AB | ABR | - | + |  |  |  |
| 58 | AB | ACR | - | + | + | + | + |
| 59 | AB | BCR | - | + | + |  |  |
| 60 | AB | ABCR | - | + |  |  |  |
| 61 | AC | AR | - | + | + |  |  |
| 62 | AC | BC | - |  |  | + |  |
| 63 | AC | BR | - | + | + | + | + |
| 64 | AC | CR | - | + | + |  |  |
| 65 | AC | ABC | - |  |  |  |  |
| 66 | AC | ABR | - | + |  | + |  |
| 67 | AC | ACR | - | + | + |  |  |
| 68 | AC | BCR | - | + | + |  |  |
| 69 | AC | ABCR | - | + |  |  |  |
| 70 | AR | BC | - | + | + |  |  |
| 71 | AR | BR | - | + | + |  |  |
| 72 | AR | CR | - | + | + |  |  |
| 73 | AR | ABC | - | + | + |  |  |
| 74 | AR | ABR | - | + | + |  |  |
| 75 | AR | ACR | - | + | + |  |  |
| 76 | AR | BCR | - | + | + |  |  |
| 77 | AR | ABCR | - | + | + |  |  |
| 78 | BC | BR | - | + | + | + | + |
| 79 | BC | CR | - | + | + | + | + |
| 80 | BC | ABC | - |  |  |  |  |
| 81 | BC | ABR | - | + |  | + |  |
| 82 | BC | ACR | - | + | + | + | + |
| 83 | BC | BCR | - | + | + |  |  |
| 84 | BC | ABCR | - | + |  |  |  |
| 85 | BR | CR | - | + | + | + | + |
| 86 | BR | ABC | - | + | + |  |  |
| 87 | BR | ABR | - | + | + |  |  |
| 88 | BR | ACR | - | + | + | + | + |
| 89 | BR | BCR | - | + | + |  |  |
| 90 | BR | ABCR | - | + | + |  |  |
| 91 | CR | ABC | - | + | + |  |  |
| 92 | CR | ABR | - | + | + | + | + |
| 93 | CR | ACR | - | + | + |  |  |
| 94 | CR | BCR | - | + | + |  |  |
| 95 | CR | ABCR | - | + | + |  |  |
| 96 | ABC | ABR | - | + |  |  |  |
| 97 | ABC | ACR | - | + | + |  |  |
| 98 | ABC | BCR | - | + | + |  |  |
| 99 | ABC | ABCR | - | + |  |  |  |
| 100 | ABR | ACR | - | + | + | + | + |
| 101 | ABR | BCR | - | + | + |  |  |
| 102 | ABR | ABCR | - | + |  |  |  |
| 103 | ACR | BCR | - | + | + |  |  |
| 104 | ACR | ABCR | - | + | + |  |  |
| 105 | BCR | ABCR | - | + | + |  |  |
| 106 | A | B | C |  |  | + |  |
| 107 | A | B | R | + | + |  |  |
| 108 | A | B | AB |  |  |  |  |
| 109 | A | B | AC |  |  | + |  |
| 110 | A | B | AR | + | + |  |  |
| 111 | A | B | BC |  |  | + |  |
| 112 | A | B | BR | + | + |  |  |
| 113 | A | B | CR | + | + | + | + |
| 114 | A | B | ABC |  |  | + |  |
| 115 | A | B | ABR | + |  |  |  |
| 116 | A | B | ACR | + | + | + | + |
| 117 | A | B | BCR | + | + | + | + |
| 118 | A | B | ABCR | + |  | + |  |
| 119 | A | C | R | + | + |  |  |
| 120 | A | C | AB |  |  | + |  |
| 121 | A | C | AC |  |  |  |  |
| 122 | A | C | AR | + | + |  |  |
| 123 | A | C | BC |  |  | + |  |
| 124 | A | C | BR | + | + | + | + |
| 125 | A | C | CR | + | + |  |  |
| 126 | A | C | ABC |  |  | + |  |
| 127 | A | C | ABR | + |  | + |  |
| 128 | A | C | ACR | + | + |  |  |
| 129 | A | C | BCR | + | + | + | + |
| 130 | A | C | ABCR | + |  | + |  |
| 131 | A | R | AB | + | + |  |  |
| 132 | A | R | AC | + | + |  |  |
| 133 | A | R | AR | + | + |  |  |
| 134 | A | R | BC | + | + |  |  |
| 135 | A | R | BR | + | + |  |  |
| 136 | A | R | CR | + | + |  |  |
| 137 | A | R | ABC | + | + |  |  |
| 138 | A | R | ABR | + | + |  |  |
| 139 | A | R | ACR | + | + |  |  |
| 140 | A | R | BCR | + | + |  |  |
| 141 | A | R | ABCR | + | + |  |  |
| 142 | A | AB | AC |  |  | + |  |
| 143 | A | AB | AR | + | + |  |  |
| 144 | A | AB | BC |  |  | + |  |
| 145 | A | AB | BR | + | + |  |  |
| 146 | A | AB | CR | + | + | + | + |
| 147 | A | AB | ABC |  |  | + |  |
| 148 | A | AB | ABR | + |  |  |  |
| 149 | A | AB | ACR | + | + | + | + |
| 150 | A | AB | BCR | + | + | + | + |
| 151 | A | AB | ABCR | + |  | + |  |
| 152 | A | AC | AR | + | + |  |  |
| 153 | A | AC | BC |  |  | + |  |
| 154 | A | AC | BR | + | + | + | + |
| 155 | A | AC | CR | + | + |  |  |
| 156 | A | AC | ABC |  |  | + |  |
| 157 | A | AC | ABR | + |  | + |  |
| 158 | A | AC | ACR | + | + |  |  |
| 159 | A | AC | BCR | + | + | + | + |
| 160 | A | AC | ABCR | + |  | + |  |
| 161 | A | AR | BC | + | + |  |  |
| 162 | A | AR | BR | + | + |  |  |
| 163 | A | AR | CR | + | + |  |  |
| 164 | A | AR | ABC | + | + |  |  |
| 165 | A | AR | ABR | + | + |  |  |
| 166 | A | AR | ACR | + | + |  |  |
| 167 | A | AR | BCR | + | + |  |  |
| 168 | A | AR | ABCR | + | + |  |  |
| 169 | A | BC | BR | + | + | + | + |
| 170 | A | BC | CR | + | + | + | + |
| 171 | A | BC | ABC |  |  |  |  |
| 172 | A | BC | ABR | + |  | + |  |
| 173 | A | BC | ACR | + | + | + | + |
| 174 | A | BC | BCR | + | + |  |  |
| 175 | A | BC | ABCR | + |  |  |  |
| 176 | A | BR | CR | + | + | + | + |
| 177 | A | BR | ABC | + | + | + | + |
| 178 | A | BR | ABR | + | + |  |  |
| 179 | A | BR | ACR | + | + | + | + |
| 180 | A | BR | BCR | + | + | + | + |
| 181 | A | BR | ABCR | + | + | + | + |
| 182 | A | CR | ABC | + | + | + | + |
| 183 | A | CR | ABR | + | + | + | + |
| 184 | A | CR | ACR | + | + |  |  |
| 185 | A | CR | BCR | + | + | + | + |
| 186 | A | CR | ABCR | + | + | + | + |
| 187 | A | ABC | ABR | + |  | + |  |
| 188 | A | ABC | ACR | + | + | + | + |
| 189 | A | ABC | BCR | + | + |  |  |
| 190 | A | ABC | ABCR | + |  |  |  |
| 191 | A | ABR | ACR | + | + | + | + |
| 192 | A | ABR | BCR | + | + | + | + |
| 193 | A | ABR | ABCR | + |  | + |  |
| 194 | A | ACR | BCR | + | + | + | + |
| 195 | A | ACR | ABCR | + | + | + | + |
| 196 | A | BCR | ABCR | + | + |  |  |
| 197 | B | C | R | + | + | + | + |
| 198 | B | C | AB |  |  | + |  |
| 199 | B | C | AC |  |  | + |  |
| 200 | B | C | AR | + | + | + | + |
| 201 | B | C | BC |  |  |  |  |
| 202 | B | C | BR | + | + | + | + |
| 203 | B | C | CR | + | + | + | + |
| 204 | B | C | ABC |  |  | + |  |
| 205 | B | C | ABR | + |  | + |  |
| 206 | B | C | ACR | + | + | + | + |
| 207 | B | C | BCR | + | + | + | + |
| 208 | B | C | ABCR | + |  | + |  |
| 209 | B | R | AB | + | + |  |  |
| 210 | B | R | AC | + | + | + | + |
| 211 | B | R | AR | + | + |  |  |
| 212 | B | R | BC | + | + | + | + |
| 213 | B | R | BR | + | + |  |  |
| 214 | B | R | CR | + | + | + | + |
| 215 | B | R | ABC | + | + | + | + |
| 216 | B | R | ABR | + | + |  |  |
| 217 | B | R | ACR | + | + | + | + |
| 218 | B | R | BCR | + | + | + | + |
| 219 | B | R | ABCR | + | + | + | + |
| 220 | B | AB | AC |  |  | + |  |
| 221 | B | AB | AR | + | + |  |  |
| 222 | B | AB | BC |  |  | + |  |
| 223 | B | AB | BR | + | + |  |  |
| 224 | B | AB | CR | + | + | + | + |
| 225 | B | AB | ABC |  |  | + |  |
| 226 | B | AB | ABR | + |  |  |  |
| 227 | B | AB | ACR | + | + | + | + |
| 228 | B | AB | BCR | + | + | + | + |
| 229 | B | AB | ABCR | + |  | + |  |
| 230 | B | AC | AR | + | + | + | + |
| 231 | B | AC | BC |  |  | + |  |
| 232 | B | AC | BR | + | + | + | + |
| 233 | B | AC | CR | + | + |  |  |
| 234 | B | AC | ABC |  |  |  |  |
| 235 | B | AC | ABR | + |  | + |  |
| 236 | B | AC | ACR | + | + |  |  |
| 237 | B | AC | BCR | + | + |  |  |
| 238 | B | AC | ABCR | + |  |  |  |
| 239 | B | AR | BC | + | + | + | + |
| 240 | B | AR | BR | + | + |  |  |
| 241 | B | AR | CR | + | + | + | + |
| 242 | B | AR | ABC | + | + | + | + |
| 243 | B | AR | ABR | + | + |  |  |
| 244 | B | AR | ACR | + | + | + | + |
| 245 | B | AR | BCR | + | + | + | + |
| 246 | B | AR | ABCR | + | + | + | + |
| 247 | B | BC | BR | + | + | + | + |
| 248 | B | BC | CR | + | + | + | + |
| 249 | B | BC | ABC |  |  | + |  |
| 250 | B | BC | ABR | + |  | + |  |
| 251 | B | BC | ACR | + | + | + | + |
| 252 | B | BC | BCR | + | + | + | + |
| 253 | B | BC | ABCR | + |  | + |  |
| 254 | B | BR | CR | + | + | + | + |
| 255 | B | BR | ABC | + | + | + | + |
| 256 | B | BR | ABR | + | + |  |  |
| 257 | B | BR | ACR | + | + | + | + |
| 258 | B | BR | BCR | + | + | + | + |
| 259 | B | BR | ABCR | + | + | + | + |
| 260 | B | CR | ABC | + | + |  |  |
| 261 | B | CR | ABR | + | + | + | + |
| 262 | B | CR | ACR | + | + |  |  |
| 263 | B | CR | BCR | + | + |  |  |
| 264 | B | CR | ABCR | + | + |  |  |
| 265 | B | ABC | ABR | + |  | + |  |
| 266 | B | ABC | ACR | + | + |  |  |
| 267 | B | ABC | BCR | + | + |  |  |
| 268 | B | ABC | ABCR | + |  |  |  |
| 269 | B | ABR | ACR | + | + | + | + |
| 270 | B | ABR | BCR | + | + | + | + |
| 271 | B | ABR | ABCR | + |  | + |  |
| 272 | B | ACR | BCR | + | + |  |  |
| 273 | B | ACR | ABCR | + | + |  |  |
| 274 | B | BCR | ABCR | + | + |  |  |
| 275 | C | R | AB | + | + | + | + |
| 276 | C | R | AC | + | + |  |  |
| 277 | C | R | AR | + | + |  |  |
| 278 | C | R | BC | + | + | + | + |
| 279 | C | R | BR | + | + | + | + |
| 280 | C | R | CR | + | + |  |  |
| 281 | C | R | ABC | + | + | + | + |
| 282 | C | R | ABR | + | + | + | + |
| 283 | C | R | ACR | + | + |  |  |
| 284 | C | R | BCR | + | + | + | + |
| 285 | C | R | ABCR | + | + | + | + |
| 286 | C | AB | AC |  |  | + |  |
| 287 | C | AB | AR | + | + | + | + |
| 288 | C | AB | BC |  |  | + |  |
| 289 | C | AB | BR | + | + |  |  |
| 290 | C | AB | CR | + | + | + | + |
| 291 | C | AB | ABC |  |  |  |  |
| 292 | C | AB | ABR | + |  |  |  |
| 293 | C | AB | ACR | + | + | + | + |
| 294 | C | AB | BCR | + | + |  |  |
| 295 | C | AB | ABCR | + |  |  |  |
| 296 | C | AC | AR | + | + |  |  |
| 297 | C | AC | BC |  |  | + |  |
| 298 | C | AC | BR | + | + | + | + |
| 299 | C | AC | CR | + | + |  |  |
| 300 | C | AC | ABC |  |  | + |  |
| 301 | C | AC | ABR | + |  | + |  |
| 302 | C | AC | ACR | + | + |  |  |
| 303 | C | AC | BCR | + | + | + | + |
| 304 | C | AC | ABCR | + |  | + |  |
| 305 | C | AR | BC | + | + | + | + |
| 306 | C | AR | BR | + | + | + | + |
| 307 | C | AR | CR | + | + |  |  |
| 308 | C | AR | ABC | + | + | + | + |
| 309 | C | AR | ABR | + | + | + | + |
| 310 | C | AR | ACR | + | + |  |  |
| 311 | C | AR | BCR | + | + | + | + |
| 312 | C | AR | ABCR | + | + | + | + |
| 313 | C | BC | BR | + | + | + | + |
| 314 | C | BC | CR | + | + | + | + |
| 315 | C | BC | ABC |  |  | + |  |
| 316 | C | BC | ABR | + |  | + |  |
| 317 | C | BC | ACR | + | + | + | + |
| 318 | C | BC | BCR | + | + | + | + |
| 319 | C | BC | ABCR | + |  | + |  |
| 320 | C | BR | CR | + | + | + | + |
| 321 | C | BR | ABC | + | + |  |  |
| 322 | C | BR | ABR | + | + |  |  |
| 323 | C | BR | ACR | + | + | + | + |
| 324 | C | BR | BCR | + | + |  |  |
| 325 | C | BR | ABCR | + | + |  |  |
| 326 | C | CR | ABC | + | + | + | + |
| 327 | C | CR | ABR | + | + | + | + |
| 328 | C | CR | ACR | + | + |  |  |
| 329 | C | CR | BCR | + | + | + | + |
| 330 | C | CR | ABCR | + | + | + | + |
| 331 | C | ABC | ABR | + |  |  |  |
| 332 | C | ABC | ACR | + | + | + | + |
| 333 | C | ABC | BCR | + | + |  |  |
| 334 | C | ABC | ABCR | + |  |  |  |
| 335 | C | ABR | ACR | + | + | + | + |
| 336 | C | ABR | BCR | + | + |  |  |
| 337 | C | ABR | ABCR | + |  |  |  |
| 338 | C | ACR | BCR | + | + | + | + |
| 339 | C | ACR | ABCR | + | + | + | + |
| 340 | C | BCR | ABCR | + | + |  |  |
| 341 | R | AB | AC | + | + | + | + |
| 342 | R | AB | AR | + | + |  |  |
| 343 | R | AB | BC | + | + | + | + |
| 344 | R | AB | BR | + | + |  |  |
| 345 | R | AB | CR | + | + | + | + |
| 346 | R | AB | ABC | + | + | + | + |
| 347 | R | AB | ABR | + | + |  |  |
| 348 | R | AB | ACR | + | + | + | + |
| 349 | R | AB | BCR | + | + | + | + |
| 350 | R | AB | ABCR | + | + | + | + |
| 351 | R | AC | AR | + | + |  |  |
| 352 | R | AC | BC | + | + | + | + |
| 353 | R | AC | BR | + | + | + | + |
| 354 | R | AC | CR | + | + |  |  |
| 355 | R | AC | ABC | + | + | + | + |
| 356 | R | AC | ABR | + | + | + | + |
| 357 | R | AC | ACR | + | + |  |  |
| 358 | R | AC | BCR | + | + | + | + |
| 359 | R | AC | ABCR | + | + | + | + |
| 360 | R | AR | BC | + | + |  |  |
| 361 | R | AR | BR | + | + |  |  |
| 362 | R | AR | CR | + | + |  |  |
| 363 | R | AR | ABC | + | + |  |  |
| 364 | R | AR | ABR | + | + |  |  |
| 365 | R | AR | ACR | + | + |  |  |
| 366 | R | AR | BCR | + | + |  |  |
| 367 | R | AR | ABCR | + | + |  |  |
| 368 | R | BC | BR | + | + | + | + |
| 369 | R | BC | CR | + | + | + | + |
| 370 | R | BC | ABC | + | + |  |  |
| 371 | R | BC | ABR | + | + | + | + |
| 372 | R | BC | ACR | + | + | + | + |
| 373 | R | BC | BCR | + | + |  |  |
| 374 | R | BC | ABCR | + | + |  |  |
| 375 | R | BR | CR | + | + | + | + |
| 376 | R | BR | ABC | + | + | + | + |
| 377 | R | BR | ABR | + | + |  |  |
| 378 | R | BR | ACR | + | + | + | + |
| 379 | R | BR | BCR | + | + | + | + |
| 380 | R | BR | ABCR | + | + | + | + |
| 381 | R | CR | ABC | + | + | + | + |
| 382 | R | CR | ABR | + | + | + | + |
| 383 | R | CR | ACR | + | + |  |  |
| 384 | R | CR | BCR | + | + | + | + |
| 385 | R | CR | ABCR | + | + | + | + |
| 386 | R | ABC | ABR | + | + | + | + |
| 387 | R | ABC | ACR | + | + | + | + |
| 388 | R | ABC | BCR | + | + |  |  |
| 389 | R | ABC | ABCR | + | + |  |  |
| 390 | R | ABR | ACR | + | + | + | + |
| 391 | R | ABR | BCR | + | + | + | + |
| 392 | R | ABR | ABCR | + | + | + | + |
| 393 | R | ACR | BCR | + | + | + | + |
| 394 | R | ACR | ABCR | + | + | + | + |
| 395 | R | BCR | ABCR | + | + |  |  |
| 396 | AB | AC | AR | + | + | + | + |
| 397 | AB | AC | BC |  |  | + |  |
| 398 | AB | AC | BR | + | + | + | + |
| 399 | AB | AC | CR | + | + | + | + |
| 400 | AB | AC | ABC |  |  | + |  |
| 401 | AB | AC | ABR | + |  | + |  |
| 402 | AB | AC | ACR | + | + | + | + |
| 403 | AB | AC | BCR | + | + | + | + |
| 404 | AB | AC | ABCR | + |  | + |  |
| 405 | AB | AR | BC | + | + | + | + |
| 406 | AB | AR | BR | + | + |  |  |
| 407 | AB | AR | CR | + | + | + | + |
| 408 | AB | AR | ABC | + | + | + | + |
| 409 | AB | AR | ABR | + | + |  |  |
| 410 | AB | AR | ACR | + | + | + | + |
| 411 | AB | AR | BCR | + | + | + | + |
| 412 | AB | AR | ABCR | + | + | + | + |
| 413 | AB | BC | BR | + | + | + | + |
| 414 | AB | BC | CR | + | + | + | + |
| 415 | AB | BC | ABC |  |  | + |  |
| 416 | AB | BC | ABR | + |  | + |  |
| 417 | AB | BC | ACR | + | + | + | + |
| 418 | AB | BC | BCR | + | + | + | + |
| 419 | AB | BC | ABCR | + |  | + |  |
| 420 | AB | BR | CR | + | + | + | + |
| 421 | AB | BR | ABC | + | + |  |  |
| 422 | AB | BR | ABR | + | + |  |  |
| 423 | AB | BR | ACR | + | + | + | + |
| 424 | AB | BR | BCR | + | + |  |  |
| 425 | AB | BR | ABCR | + | + |  |  |
| 426 | AB | CR | ABC | + | + | + | + |
| 427 | AB | CR | ABR | + | + | + | + |
| 428 | AB | CR | ACR | + | + | + | + |
| 429 | AB | CR | BCR | + | + | + | + |
| 430 | AB | CR | ABCR | + | + | + | + |
| 431 | AB | ABC | ABR | + |  |  |  |
| 432 | AB | ABC | ACR | + | + | + | + |
| 433 | AB | ABC | BCR | + | + |  |  |
| 434 | AB | ABC | ABCR | + |  |  |  |
| 435 | AB | ABR | ACR | + | + | + | + |
| 436 | AB | ABR | BCR | + | + |  |  |
| 437 | AB | ABR | ABCR | + |  |  |  |
| 438 | AB | ACR | BCR | + | + | + | + |
| 439 | AB | ACR | ABCR | + | + | + | + |
| 440 | AB | BCR | ABCR | + | + |  |  |
| 441 | AC | AR | BC | + | + | + | + |
| 442 | AC | AR | BR | + | + | + | + |
| 443 | AC | AR | CR | + | + |  |  |
| 444 | AC | AR | ABC | + | + | + | + |
| 445 | AC | AR | ABR | + | + | + | + |
| 446 | AC | AR | ACR | + | + |  |  |
| 447 | AC | AR | BCR | + | + | + | + |
| 448 | AC | AR | ABCR | + | + | + | + |
| 449 | AC | BC | BR | + | + | + | + |
| 450 | AC | BC | CR | + | + | + | + |
| 451 | AC | BC | ABC |  |  | + |  |
| 452 | AC | BC | ABR | + |  | + |  |
| 453 | AC | BC | ACR | + | + | + | + |
| 454 | AC | BC | BCR | + | + | + | + |
| 455 | AC | BC | ABCR | + |  | + |  |
| 456 | AC | BR | CR | + | + | + | + |
| 457 | AC | BR | ABC | + | + | + | + |
| 458 | AC | BR | ABR | + | + | + | + |
| 459 | AC | BR | ACR | + | + | + | + |
| 460 | AC | BR | BCR | + | + | + | + |
| 461 | AC | BR | ABCR | + | + | + | + |
| 462 | AC | CR | ABC | + | + |  |  |
| 463 | AC | CR | ABR | + | + | + | + |
| 464 | AC | CR | ACR | + | + |  |  |
| 465 | AC | CR | BCR | + | + |  |  |
| 466 | AC | CR | ABCR | + | + |  |  |
| 467 | AC | ABC | ABR | + |  |  |  |
| 468 | AC | ABC | ACR | + | + |  |  |
| 469 | AC | ABC | BCR | + | + |  |  |
| 470 | AC | ABC | ABCR | + |  |  |  |
| 471 | AC | ABR | ACR | + | + | + | + |
| 472 | AC | ABR | BCR | + | + | + | + |
| 473 | AC | ABR | ABCR | + |  | + |  |
| 474 | AC | ACR | BCR | + | + |  |  |
| 475 | AC | ACR | ABCR | + | + |  |  |
| 476 | AC | BCR | ABCR | + | + |  |  |
| 477 | AR | BC | BR | + | + | + | + |
| 478 | AR | BC | CR | + | + | + | + |
| 479 | AR | BC | ABC | + | + |  |  |
| 480 | AR | BC | ABR | + | + | + | + |
| 481 | AR | BC | ACR | + | + | + | + |
| 482 | AR | BC | BCR | + | + |  |  |
| 483 | AR | BC | ABCR | + | + |  |  |
| 484 | AR | BR | CR | + | + | + | + |
| 485 | AR | BR | ABC | + | + | + | + |
| 486 | AR | BR | ABR | + | + |  |  |
| 487 | AR | BR | ACR | + | + | + | + |
| 488 | AR | BR | BCR | + | + | + | + |
| 489 | AR | BR | ABCR | + | + | + | + |
| 490 | AR | CR | ABC | + | + | + | + |
| 491 | AR | CR | ABR | + | + | + | + |
| 492 | AR | CR | ACR | + | + |  |  |
| 493 | AR | CR | BCR | + | + | + | + |
| 494 | AR | CR | ABCR | + | + | + | + |
| 495 | AR | ABC | ABR | + | + | + | + |
| 496 | AR | ABC | ACR | + | + | + | + |
| 497 | AR | ABC | BCR | + | + |  |  |
| 498 | AR | ABC | ABCR | + | + |  |  |
| 499 | AR | ABR | ACR | + | + | + | + |
| 500 | AR | ABR | BCR | + | + | + | + |
| 501 | AR | ABR | ABCR | + | + | + | + |
| 502 | AR | ACR | BCR | + | + | + | + |
| 503 | AR | ACR | ABCR | + | + | + | + |
| 504 | AR | BCR | ABCR | + | + |  |  |
| 505 | BC | BR | CR | + | + | + | + |
| 506 | BC | BR | ABC | + | + | + | + |
| 507 | BC | BR | ABR | + | + | + | + |
| 508 | BC | BR | ACR | + | + | + | + |
| 509 | BC | BR | BCR | + | + | + | + |
| 510 | BC | BR | ABCR | + | + | + | + |
| 511 | BC | CR | ABC | + | + | + | + |
| 512 | BC | CR | ABR | + | + | + | + |
| 513 | BC | CR | ACR | + | + | + | + |
| 514 | BC | CR | BCR | + | + | + | + |
| 515 | BC | CR | ABCR | + | + | + | + |
| 516 | BC | ABC | ABR | + |  | + |  |
| 517 | BC | ABC | ACR | + | + | + | + |
| 518 | BC | ABC | BCR | + | + |  |  |
| 519 | BC | ABC | ABCR | + |  |  |  |
| 520 | BC | ABR | ACR | + | + | + | + |
| 521 | BC | ABR | BCR | + | + | + | + |
| 522 | BC | ABR | ABCR | + |  | + |  |
| 523 | BC | ACR | BCR | + | + | + | + |
| 524 | BC | ACR | ABCR | + | + | + | + |
| 525 | BC | BCR | ABCR | + | + |  |  |
| 526 | BR | CR | ABC | + | + | + | + |
| 527 | BR | CR | ABR | + | + | + | + |
| 528 | BR | CR | ACR | + | + | + | + |
| 529 | BR | CR | BCR | + | + | + | + |
| 530 | BR | CR | ABCR | + | + | + | + |
| 531 | BR | ABC | ABR | + | + |  |  |
| 532 | BR | ABC | ACR | + | + | + | + |
| 533 | BR | ABC | BCR | + | + |  |  |
| 534 | BR | ABC | ABCR | + | + |  |  |
| 535 | BR | ABR | ACR | + | + | + | + |
| 536 | BR | ABR | BCR | + | + |  |  |
| 537 | BR | ABR | ABCR | + | + |  |  |
| 538 | BR | ACR | BCR | + | + | + | + |
| 539 | BR | ACR | ABCR | + | + | + | + |
| 540 | BR | BCR | ABCR | + | + |  |  |
| 541 | CR | ABC | ABR | + | + | + | + |
| 542 | CR | ABC | ACR | + | + |  |  |
| 543 | CR | ABC | BCR | + | + |  |  |
| 544 | CR | ABC | ABCR | + | + |  |  |
| 545 | CR | ABR | ACR | + | + | + | + |
| 546 | CR | ABR | BCR | + | + | + | + |
| 547 | CR | ABR | ABCR | + | + | + | + |
| 548 | CR | ACR | BCR | + | + |  |  |
| 549 | CR | ACR | ABCR | + | + |  |  |
| 550 | CR | BCR | ABCR | + | + |  |  |
| 551 | ABC | ABR | ACR | + | + | + | + |
| 552 | ABC | ABR | BCR | + | + |  |  |
| 553 | ABC | ABR | ABCR | + |  |  |  |
| 554 | ABC | ACR | BCR | + | + |  |  |
| 555 | ABC | ACR | ABCR | + | + |  |  |
| 556 | ABC | BCR | ABCR | + | + |  |  |
| 557 | ABR | ACR | BCR | + | + | + | + |
| 558 | ABR | ACR | ABCR | + | + | + | + |
| 559 | ABR | BCR | ABCR | + | + |  |  |
| 560 | ACR | BCR | ABCR | + | + |  |  |
| **total** | | | | **504** | **440** | **299** | **235** |
