# Supplementary material for: Allele Intersection Analysis: A Novel Tool for Multi Locus Sequence Assignment in Multiply Infected Hosts
Source: PLoS One. 2011 Jul 15;6(7):e22198. doi: 10.1371/journal.pone.0022198 (PMC3137623; doi:10.1371/journal.pone.0022198)
Supplement: Table S2 — Observed numbers of plasmids cloned from DNA of a quadruply infected host Neufeld, and expected numbers based on each strain's relative load in the original DNA extract. (DOC) [file pone.0022198.s004.doc]

**Table S2.**

Observed numbers of plasmids cloned from DNA of a quadruply infected host Neufeld, and expected numbers based on each strain’s relative load in the original DNA extract.

| **Locus** | **Primer** | **n total *^a^*** | **arte-fact *^b^*** | ***w*Cer1 (n)** | | ***w*Cer2 (n)** | | ***w*Cer4 (n)** | | ***w*Cer5 (n)** | |
| --- | --- | --- | --- | --- | --- | --- | --- | --- | --- | --- | --- |
|  |  |  |  | **obs.** | **exp.** | **obs.** | **exp.** | **obs.** | **exp.** | **obs.** | **exp.** |
| ***wsp*** | standard | 13 | 4 | 4 | 2.17 | 5 | 4.43 | 0 | 1.99 | 0 | 0.00 |
|  | B-group | 11 | 0 | 0 | 0.00 | 0 | 0.00 | 0 | 0.00 | 11 | 11.00 |
| ***ftsZ*** | standard | 16 | 0 | 5 | 3.86 | 5 | 7.87 | 6 | 3.54 | 0 | 0.00 |
|  | B-group | 11 | 0 | 0 | 0.00 | 0 | 0.00 | 0 | 0.00 | 11 | 11.00 |
| ***gatB*** | standard | 10 | 0 | 3 | 2.41 | 5 | 4.92 | 2 | 2.21 | 0 | 0.00 |
|  | B-group | 11 | 0 | 0 | 0.00 | 0 | 0.00 | 0 | 0.00 | 11 | 11.00 |
| ***hcpA*** | standard | 14 | 0 | 5 | 3.37 | 9 | 6.89 | 0 | 3.09 | 0 | 0.00 |
|  | B-group | 12 | 0 | 0 | 0.00 | 0 | 0.00 | 0 | 0.00 | 12 | 12.00 |
| ***fbpA*** | standard | 12 | 3 | 7 | 2.17 | 2 | 4.43 | 0 | 1.99 | 0 | 0.00 |
|  | B-group | 14 | 2 | 3 | 0.00 | 1 | 0.00 | 2 | 0.00 | 6 | 12.00 |
| ***coxA*** | standard | 16 | 00 | =*w*Cer4 | | 9 | 7.87 | 7 | 3.54 | 0 | 0.00 |
|  | B-group | 8 |  |  |  | 0 | 0.00 | 0 | 0.00 | 8 | 8.00 |

***^a^*** After *wsp* and MLST PCR, amplicons were cloned, 16 colonies per ligation were picked and plasmids with correct insert length subjected to sequencing.

*^b^ in vitro* recombinations not reproducible in repeated PCR reactions
