# Supplementary material for: Allele Intersection Analysis: A Novel Tool for Multi Locus Sequence Assignment in Multiply Infected Hosts
Source: PLoS One. 2011 Jul 15;6(7):e22198. doi: 10.1371/journal.pone.0022198 (PMC3137623; doi:10.1371/journal.pone.0022198)
Supplement: Table S3 — All possible 3- and 4-fold combinations of infection types found in R. cerasi field samples. These is a total of 126 combinations, of which 81 are informative. Uninformative combinations are shaded. (DOC) [file pone.0022198.s005.doc]

**Table S3**

All possible 3- and 4-fold combinations of infection types found in *R. cerasi* field samples. These is a total of 126 combinations, of which 81 are informative. Uninformative combinations are shaded.

| Infection types | | | | Informative ? |
| --- | --- | --- | --- | --- |
| Individual 1 | Individual 2 | Individual 3 | Individual 4 |
| 1 | 1+2 | 1+4 | - | no |
| 1 | 1+2 | 1+5 | - | no |
| 1 | 1+2 | 1+2+4 | - | no |
| 1 | 1+2 | 1+2+5 | - | no |
| 1 | 1+2 | 1+4+5 | - | no |
| 1 | 1+2 | 1+2+4+5 | - | no |
| 1 | 1+4 | 1+5 | - | no |
| 1 | 1+4 | 1+2+4 | - | no |
| 1 | 1+4 | 1+2+5 | - | no |
| 1 | 1+4 | 1+4+5 | - | no |
| 1 | 1+4 | 1+2+4+5 | - | no |
| 1 | 1+5 | 1+2+4 | - | no |
| 1 | 1+5 | 1+2+5 | - | no |
| 1 | 1+5 | 1+4+5 | - | no |
| 1 | 1+5 | 1+2+4+5 | - | no |
| 1 | 1+2+4 | 1+2+5 | - | yes |
| 1 | 1+2+4 | 1+4+5 | - | yes |
| 1 | 1+2+4 | 1+2+4+5 | - | no |
| 1 | 1+2+5 | 1+4+5 | - | yes |
| 1 | 1+2+5 | 1+2+4+5 | - | no |
| 1 | 1+4+5 | 1+2+4+5 | - | no |
| 1+2 | 1+4 | 1+5 | - | yes |
| 1+2 | 1+4 | 1+2+4 | - | no |
| 1+2 | 1+4 | 1+2+5 | - | yes |
| 1+2 | 1+4 | 1+4+5 | - | yes |
| 1+2 | 1+4 | 1+2+4+5 | - | yes |
| 1+2 | 1+5 | 1+2+4 | - | yes |
| 1+2 | 1+5 | 1+2+5 | - | no |
| 1+2 | 1+5 | 1+4+5 | - | yes |
| 1+2 | 1+5 | 1+2+4+5 | - | yes |
| 1+2 | 1+2+4 | 1+2+5 | - | no |
| 1+2 | 1+2+4 | 1+4+5 | - | yes |
| 1+2 | 1+2+4 | 1+2+4+5 | - | no |
| 1+2 | 1+2+5 | 1+4+5 | - | yes |
| 1+2 | 1+2+5 | 1+2+4+5 | - | no |
| 1+2 | 1+4+5 | 1+2+4+5 | - | no |
| 1+4 | 1+5 | 1+2+4 | - | yes |
| 1+4 | 1+5 | 1+2+5 | - | yes |
| 1+4 | 1+5 | 1+4+5 | - | no |
| 1+4 | 1+5 | 1+2+4+5 | - | yes |
| 1+4 | 1+2+4 | 1+2+5 | - | yes |
| 1+4 | 1+2+4 | 1+4+5 | - | no |
| 1+4 | 1+2+4 | 1+2+4+5 | - | no |
| 1+4 | 1+2+5 | 1+4+5 | - | yes |
| 1+4 | 1+2+5 | 1+2+4+5 | - | no |
| 1+4 | 1+4+5 | 1+2+4+5 | - | no |
| 1+5 | 1+2+4 | 1+2+5 | - | yes |
| 1+5 | 1+2+4 | 1+4+5 | - | yes |
| 1+5 | 1+2+4 | 1+2+4+5 | - | no |
| 1+5 | 1+2+5 | 1+4+5 | - | no |
| 1+5 | 1+2+5 | 1+2+4+5 | - | no |
| 1+5 | 1+4+5 | 1+2+4+5 | - | no |
| 1+2+4 | 1+2+5 | 1+4+5 | - | yes |
| 1+2+4 | 1+2+5 | 1+2+4+5 | - | no |
| 1+2+4 | 1+4+5 | 1+2+4+5 | - | no |
| 1+2+5 | 1+4+5 | 1+2+4+5 | - | no |
| 1 | 1+2 | 1+4 | 1+5 | yes |
| 1 | 1+2 | 1+4 | 1+2+4 | no |
| 1 | 1+2 | 1+4 | 1+2+5 | yes |
| 1 | 1+2 | 1+4 | 1+4+5 | yes |
| 1 | 1+2 | 1+4 | 1+2+4+5 | yes |
| 1 | 1+2 | 1+5 | 1+2+4 | yes |
| 1 | 1+2 | 1+5 | 1+2+5 | no |
| 1 | 1+2 | 1+5 | 1+4+5 | yes |
| 1 | 1+2 | 1+5 | 1+2+4+5 | yes |
| 1 | 1+2 | 1+2+4 | 1+2+5 | yes |
| 1 | 1+2 | 1+2+4 | 1+4+5 | yes |
| 1 | 1+2 | 1+2+4 | 1+2+4+5 | yes |
| 1 | 1+2 | 1+2+5 | 1+4+5 | yes |
| 1 | 1+2 | 1+2+5 | 1+2+4+5 | yes |
| 1 | 1+2 | 1+4+5 | 1+2+4+5 | no |
| 1 | 1+4 | 1+5 | 1+2+4 | yes |
| 1 | 1+4 | 1+5 | 1+2+5 | yes |
| 1 | 1+4 | 1+5 | 1+4+5 | no |
| 1 | 1+4 | 1+5 | 1+2+4+5 | yes |
| 1 | 1+4 | 1+2+4 | 1+2+5 | yes |
| 1 | 1+4 | 1+2+4 | 1+4+5 | yes |
| 1 | 1+4 | 1+2+4 | 1+2+4+5 | yes |
| 1 | 1+4 | 1+2+5 | 1+4+5 | yes |
| 1 | 1+4 | 1+2+5 | 1+2+4+5 | no |
| 1 | 1+4 | 1+4+5 | 1+2+4+5 | yes |
| 1 | 1+5 | 1+2+4 | 1+2+5 | yes |
| 1 | 1+5 | 1+2+4 | 1+4+5 | yes |
| 1 | 1+5 | 1+2+4 | 1+2+4+5 | no |
| 1 | 1+5 | 1+2+5 | 1+4+5 | yes |
| 1 | 1+5 | 1+2+5 | 1+2+4+5 | yes |
| 1 | 1+5 | 1+4+5 | 1+2+4+5 | yes |
| 1 | 1+2+4 | 1+2+5 | 1+4+5 | yes |
| 1 | 1+2+4 | 1+2+5 | 1+2+4+5 | yes |
| 1 | 1+2+4 | 1+4+5 | 1+2+4+5 | yes |
| 1 | 1+2+5 | 1+4+5 | 1+2+4+5 | yes |
| 1+2 | 1+4 | 1+5 | 1+2+4 | yes |
| 1+2 | 1+4 | 1+5 | 1+2+5 | yes |
| 1+2 | 1+4 | 1+5 | 1+4+5 | yes |
| 1+2 | 1+4 | 1+5 | 1+2+4+5 | yes |
| 1+2 | 1+4 | 1+2+4 | 1+2+5 | yes |
| 1+2 | 1+4 | 1+2+4 | 1+4+5 | yes |
| 1+2 | 1+4 | 1+2+4 | 1+2+4+5 | yes |
| 1+2 | 1+4 | 1+2+5 | 1+4+5 | yes |
| 1+2 | 1+4 | 1+2+5 | 1+2+4+5 | yes |
| 1+2 | 1+4 | 1+4+5 | 1+2+4+5 | yes |
| 1+2 | 1+5 | 1+2+4 | 1+2+5 | yes |
| 1+2 | 1+5 | 1+2+4 | 1+4+5 | yes |
| 1+2 | 1+5 | 1+2+4 | 1+2+4+5 | yes |
| 1+2 | 1+5 | 1+2+5 | 1+4+5 | yes |
| 1+2 | 1+5 | 1+2+5 | 1+2+4+5 | yes |
| 1+2 | 1+5 | 1+4+5 | 1+2+4+5 | yes |
| 1+2 | 1+2+4 | 1+2+5 | 1+4+5 | yes |
| 1+2 | 1+2+4 | 1+2+5 | 1+2+4+5 | no |
| 1+2 | 1+2+4 | 1+4+5 | 1+2+4+5 | yes |
| 1+2 | 1+2+5 | 1+4+5 | 1+2+4+5 | yes |
| 1+4 | 1+5 | 1+2+4 | 1+2+5 | yes |
| 1+4 | 1+5 | 1+2+4 | 1+4+5 | yes |
| 1+4 | 1+5 | 1+2+4 | 1+2+4+5 | yes |
| 1+4 | 1+5 | 1+2+5 | 1+4+5 | yes |
| 1+4 | 1+5 | 1+2+5 | 1+2+4+5 | yes |
| 1+4 | 1+5 | 1+4+5 | 1+2+4+5 | yes |
| 1+4 | 1+2+4 | 1+2+5 | 1+4+5 | yes |
| 1+4 | 1+2+4 | 1+2+5 | 1+2+4+5 | yes |
| 1+4 | 1+2+4 | 1+4+5 | 1+2+4+5 | no |
| 1+4 | 1+2+5 | 1+4+5 | 1+2+4+5 | yes |
| 1+5 | 1+2+4 | 1+2+5 | 1+4+5 | yes |
| 1+5 | 1+2+4 | 1+2+5 | 1+2+4+5 | yes |
| 1+5 | 1+2+4 | 1+4+5 | 1+2+4+5 | yes |
| 1+5 | 1+2+5 | 1+4+5 | 1+2+4+5 | no |
| 1+2+4 | 1+2+5 | 1+4+5 | 1+2+4+5 | yes |
