# Supplementary material for: Allele Intersection Analysis: A Novel Tool for Multi Locus Sequence Assignment in Multiply Infected Hosts
Source: PLoS One. 2011 Jul 15;6(7):e22198. doi: 10.1371/journal.pone.0022198 (PMC3137623; doi:10.1371/journal.pone.0022198)
Supplement: Glossary S1 — Short descriptions of set therory terms used in this paper. (DOC) [file pone.0022198.s008.doc]

**Glossary**

Set

A set is a collection of distinct objects, called elements. In mathematics, the elements of a set can be anything: numbers, colours, letters, other sets, etc. Often sets are denoted with capital letters. Sets S and T are equal if and only if they contain precisely the same elements. A set can be described by listing its elements in braces: S = {red, green, blue}.

Cardinality

The cardinality |S| of a set S is the number of elements of this particular set. A set with only one member has the cardinality of 1. The empty set {} has the cardinality of zero.

Intersection

The intersection of two sets S and T, denoted by S ∩ T, is the set of all elements which are members of both S and T: if S = {red, green} and T = {green, blue}, the intersection S ∩ T = {green}.

Complement

The complement of T in S, denoted by S \ T, is the set of all elements which are members of S but not members of T. In the above sample, S \ T = {red}. Different to the arithmetic subtraction, it is valid to ‘subtract’ an element from a set that is not member of the set, leaving the original set unchanged. Note that S \ T ≠ T \ S.

Infection type

The infection type is the combination of *Wolbachia* strains infecting one particular insect. It can be interpreted as a set, with the different *Wolbachia* strains as its elements. In a species harbouring *n* *Wolbachia* strains, different individuals may have different infection types, which will all have cardinalities ≤ *n*. The infection type will resemble the sum of different *Wolbachia* sequences cloned from the same locus of the same individual.

Type combination

A type combination is a set. Its elements are different infection types, which were sampled from different individuals of the same species.

Allele Intersection Analysis (AIA)

AIA is the attempt to dissect the elements of a type combination into numerous sets with a cardinality of 1 by repeatedly applying intersection and complement operations. A type combination allowing such a complete dissection is called informative. Informative type combinations can be used to identify *Wolbachia* MLST profiles in multiply infected species without the need of physically segregating single *Wolbachia* strains e.g. through host breeding under environmental stress, artificial transfer through microinjection or cell culturing.
